# Genome Editing of *eIF4E1* in Tomato Confers Resistance to Pepper Mottle Virus

**DOI:** 10.3389/fpls.2020.01098

**Published:** 2020-07-24

**Authors:** Yoo-Joung Yoon, Jelli Venkatesh, Joung-Ho Lee, Jinhee Kim, Hye-Eun Lee, Do-Sun Kim, Byoung-Cheorl Kang

**Affiliations:** ^1^ Department of Plant Science and Plant Genomics and Breeding Institute, College of Agriculture and Life Sciences, Seoul National University, Seoul, South Korea; ^2^ Vegetable Research Division, National Institute of Horticultural and Herbal Science, RDA, Jeonju-si, South Korea

**Keywords:** CRISPR/Cas9, eukaryotic translation initiation factor 4E (*eIF4E*), genome editing, potyvirus, phytoene desaturase (*PDS*), *Solanum lycopersicum* cv. Micro-Tom

## Abstract

Many of the recessive virus-resistance genes in plants encode eukaryotic translation initiation factors (eIFs), including eIF4E, eIF4G, and related proteins. Notably, *eIF4E* and its isoform *eIF(iso)4E* are pivotal for viral infection and act as recessive resistance genes against various potyviruses in a wide range of plants. In this study, we used Clustered Regularly Interspaced Palindromic Repeats/CRISPR-associated protein 9 (CRISPR/Cas9)-mediated targeted mutagenesis to test whether novel sequence-specific mutations at *eIF4E1* in *Solanum lycopersicum* (tomato) cv. Micro-Tom could confer enhanced resistance to potyviruses. This approach produced heritable homozygous mutations in the transgene-free E_1_ generation. Sequence analysis of *eIF4E1* from E_0_ transgenic plants expressing *Cas9* and *eIF4E-sgRNA* transcripts identified chimeric deletions ranging from 11 to 43 bp. Genotype analysis of the *eIF4E1*-edited lines in E_0_, E_1_, and E_2_ transgenic tomato plants showed that the mutations were transmitted to subsequent generations. When homozygous mutant lines were tested for resistance to potyviruses, they exhibited no resistance to tobacco etch virus (TEV). Notably, however, several mutant lines showed no accumulation of viral particles upon infection with pepper mottle virus (PepMoV). These results indicate that site-specific mutation of tomato *eIF4E1* successfully conferred enhanced resistance to PepMoV. Thus, this study demonstrates the feasibility of the use of CRISPR/Cas9 approach to accelerate breeding for trait improvement in tomato plants.

## Introduction

*Potyviridae* is the largest family of plant RNA viruses, which account for about 30% of known plant viruses, cause considerable damage to crop plants (Ward and Shukla, 1991; Cui and Wang, 2019). The potyviruses tobacco etch virus (TEV), potato virus Y (PVY), chilli veinal mottle virus (ChiVMV), pepper mottle virus (PepMoV), and pepper veinal mottle virus (PVMV) infect numerous solanaceous plants, including tomato, potato, and pepper (Kothari et al., 2010; Zhao et al., 2014; Hančinský et al., 2020). Potyviruses, such as PepMoV and TEV are potential threat to tomato crop (Wintermantel, 2011; Melzer et al., 2012).

Eukaryotic translation initiation factor (eIF) genes, such as eukaryotic translation initiation factor 4E (eIF4E), eukaryotic translation initiation factor (Iso) 4E (eIF(iso)4E), and eukaryotic translation initiation factor 4G (eIF4G), are required for RNA viruses to maintain their lifecycle (Sanfacon, 2015; Revers and García, 2015). The number of eIF4E family members is species-dependent. In tomato, the eIF4E family consists of two *eIF4E* homologs (*eIF4E1* and *eIF4E2*), one *eIF(iso)*4E homolog (Lebaron et al., 2016). Notably, several eIF genes confer recessive resistance to one or more potyviruses in Solanaceae crops (Kang et al., 2005a; Ruffel et al., 2006; Kang et al., 2007; Lee et al., 2013). A single recessive resistance locus *pot-1* encoding eIF4E1 protein was identified from the tomato wild relative, *Solanum habrochaites* accession PI247087. *eIF4E1* confers resistance to several potyviruses, including PVY, TEV, and PepMoV (Ruffel et al., 2005; Kang et al., 2005a; Wang, 2015). Knockout mutants of tomato *eIF4E2* and *eIF(iso)4E* were reported to be fully susceptible to potyviruses, thus suggesting prominent role of eIF4E1 in potyviral resistance in Solanaceous crops (Robaglia and Caranta, 2006; Charron et al., 2008).

The eIF protein eIF4E is a component of a multiprotein complex that aids the initiation of protein translation by enabling recognition and interaction with the mRNA cap structure and recruitment of ribosomes. The viral genome-linked protein (VPg) of potyviruses is covalently connected to the 5′ end of viral RNA and acts as an analog of the eukaryotic mRNA cap structure during protein translation (Wang, 2015). The physical interaction between the host factor, eIF4E (or its homolog eIF(iso)4E), and VPg is crucial for potyvirus infectivity (Miyoshi et al., 2006; Robaglia and Caranta, 2006; Hwang et al., 2009; Kim et al., 2013; Kim et al., 2014; Tavert-Roudet et al., 2017; de Oliveira et al., 2019). Moreover, mutations in these host factors can inhibit the interaction between the host factor and the VPg, and consequently inhibit viral proliferation and host infection (Duprat et al., 2002; Lellis et al., 2002; Sato et al., 2005; Rodríguez-Hernández et al., 2012).

The majority of naturally occurring plant recessive resistance genes have been mapped to mutations in genes encoding the isoforms of the translation initiation factors eIF4E and eIF4G that hinder their interactions with viral RNAs or proteins (Kang et al., 2005a; Ruffel et al., 2006; Le Gall et al., 2011; Wang and Krishnaswamy, 2012; Lee et al., 2013). Natural recessive resistance to potyviruses has been exploited in numerous breeding programs (Kang et al., 2005b; Ruffel et al., 2006; Lee et al., 2013). However, conventional breeding requires massive backcrossing to introgress the trait of interest into an elite background; furthermore, the availability of favorable alleles in natural populations is limited. New alleles can be created by random mutagenesis (Nicaise, 2014), but this requires labor-intensive, time-consuming screening of large populations to select mutants with desirable properties.

Advances in genome-editing tools have accelerated site-directed mutagenesis in crops. Clustered regularly interspersed palindromic repeats (CRISPR)/CRISPR-associated protein 9 (CRISPR/Cas9) is a targeted genome-editing technique derived from the adaptive immune mechanism of *Staphylococcus pyogenes* against bacteriophages (Hsu et al., 2014). Since its first report in 2012, CRISPR/Cas9 has become the technology of choice for genome editing due to its ease, low cost, and significantly shorter timeframe for construct preparation compared to those of other genome-editing tools, such as zinc finger nucleases (ZFN) and transcription activator-like effector nucleases (TALENs) (Cong and Zhang, 2015). CRISPR/Cas9 has been utilized for site-directed mutagenesis in microbes, animals, human cells, and plants (Jiang et al., 2013; Wu et al., 2013; Zhang et al., 2017; Shapiro et al., 2018). Precise genome editing of host factors can be deployed for development of recessive genetic resistance against viral diseases in plants (Wang, 2015). However, the application of CRISPR/Cas9 tools to improve plant resistance to pathogens has not been widely explored, with only a few reports to date (Chandrasekaran et al., 2016; Pyott et al., 2016; Wang et al., 2016; Peng et al., 2017; Gomez et al., 2019). Recently, CRISPR/Cas9 genome editing of plants for potyvirus resistance has been reported. For instance, knockout of the gene *eIF(iso)4E* in the model crop plant *Arabidopsis thaliana* was shown to confer resistance to turnip mosaic virus (TuMV) (Pyott et al., 2016). In another study, CRISPR/Cas9-mediated knockout of *eIF4E* in cucumber resulted in broad-spectrum viral resistance to several plant viruses, including cucumber vein yellowing virus (CVYV), zucchini yellow mosaic virus (ZYMV), and papaya ringspot mosaic virus-w (PRSV-W) (Chandrasekaran et al., 2016). Similarly novel allelic variants of rice *eIF4G* generated through CRISPR/Cas9 mediated genome editing conferred resistance to rice tungro spherical virus (Macovei et al., 2018). Thus, targeted genome editing can be expected to accelerate plant breeding for disease-resistant crop plants by facilitating the introduction of precise and predictable genetic changes directly into an elite strain background.

In this study, to introduce allelic variations in the *eIF4E* gene, we mutated *eIF4E1* in the tomato cultivar Micro-Tom using CRISPR/Cas9 technology and *Agrobacterium*-mediated transformation, and bred the E_0_ transgenic plants carrying *eIF4E1* mutations to produce E_1_ and E_2_ progeny. We then tested the resistance of the homozygous mutant lines to the potyviruses TEV and PepMoV. The homozygous Micro-Tom mutant lines carrying genome-edited *eIF4E1* were resistant to PepMoV, although not to TEV, and showed normal plant growth and development after PepMoV challenge. Furthermore, by segregation in the E_1_ generation, we were able to select virus-resistant plants that carried an edited *eIF4E1* gene or genes but not the introduced transgene. Altogether, this study thus provides important information for understanding and analyzing tomato CRISPR/Cas9 mutants, and accelerating breeding for trait improvement.

## Materials and Methods

### Plant Materials and Growth Conditions

Seeds of *Solanum lycopersicum* cv. Micro-Tom were surface sterilized in 70% ethanol for 1 min and in 2% NaOCl with one drop of Tween 20 for 15 min, and then rinsed four or five times with sterilized water. Seeds were germinated on 1/2 MS medium (Murashige and Skoog, 1962) containing 20 g/L sucrose and 8 g/L agar agar. All cultures were grown at 24°C with a 16 h light/8 h dark cycle under cool fluorescent light. Cotyledons of 7–8-day-old seedlings, before the appearance of the first true leaves, were used as explants for tissue culture.

### Single Guide RNA (sgRNA) Design and Vector Construction

**Figure 1:**
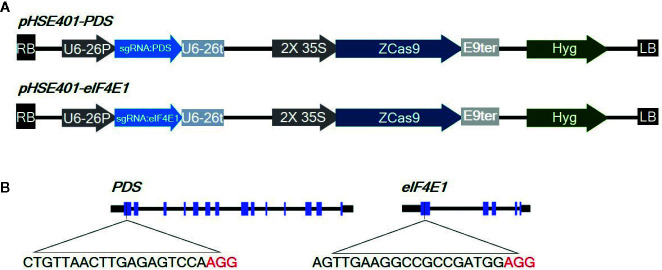
Diagrams of the CRISPR/Cas9 vector constructs and sgRNA target sites **(A)** Diagram of the cassettes (pHSE401-PDS and pHSE401-eIF4E1) expressing the maize codon-optimized *Cas9* gene under the control of the 35S promoter, and the PDS- and eIF4E-sgRNA sequence driven by the Arabidopsis U6-26 polymerase III promoter. **(B)** Diagram of the CRISPR/Cas9 target sites within *PDS* and *eIF4E1*. The sgRNA target sequences are shown in black letters, followed by protospacer-adjacent motif (PAM) sequences in red.

eIF4E is a plant cellular translation initiation factor essential for potyvirus infection, and mutations in the *eIF4E* gene can confer resistance to potyviruses. In this study, we aimed to use CRISPR/Cas9-mediated targeted genome editing of *eIF4E1* (GenBank: AY723733) to develop potyvirus-resistant tomato plants. The gene *phytoene desaturase* (*PDS*) (GenBank: EF650011), encoding the key enzyme in carotenoid biosynthesis, was used as a control gene to test genome-editing efficiency due to the easily detectable photobleached phenotype of *PDS* mutants. To design sgRNAs, we identified appropriate target sgRNA sequences using the CCTop - CRISPR/Cas9 target online predictor (https://crispr.cos.uni-heidelberg.de/index.html). sgRNAs targeting the first exon with high prediction scores were used for genome editing (**Figure 1**), since mutations in the 5′ region or first exon would increase the chance of creating nonfunctional proteins by causing frameshifts or early stop codons. These sgRNAs were cloned under the control of the AtU6-26 promoter into a binary vector (pHSE401) carrying a maize codon-optimized *Cas9* gene driven by the CaMV 35S promoter. The 23-bp sequences of the corresponding primers, PDSgRNA_F and PDSgRNA_R (or eIF4E1gRNA_F and eIF4E1gRNA_R), flanked by a *Bsa*I recognition site, were annealed and cloned into a *Bsa*I site in the vector pHSE401 by Golden Gate cloning according to a previously reported method (Xing et al., 2014). CRISPR/Cas9 vectors were transformed into *Agrobacterium tumefaciens* strain GV3101 by electroporation.

### *Agrobacterium*-Mediated Tomato Transformation

A single *Agrobacterium* colony was inoculated into 15 mL of liquid LB medium containing 50 μg/mL kanamycin and 50 μg/mL rifampicin and incubated in a shaking incubator at 28°C for 12–16 h until it reached an OD_600_ of 0.6. The *Agrobacterium* suspension was then centrifuged at 8,000 rpm for 10 min at 20°C to collect the pellet. The pellet was completely resuspended in liquid 1/2 MS medium containing 3% sucrose and 200 μM acetosyringone. Cotyledons from 7–8-day-old seedlings were excised under sterile conditions, and then the tip of each cotyledon was removed, sectioned transversely into two fragments, and incubated adaxial side down in a preculture medium consisting of MS with 30 g/L sucrose, 1 mg/L 1-naphthaleneacetic acid (NAA), 1 mg/L benzylaminopurine (BAP) for 2 days, and 8 g/L agar agar. Explants were co-cultured in the *Agrobacterium* suspension for 20 min, transferred to sterile filter paper to briefly drain excess suspension, and then placed on the same medium used for preculture and incubated for 2 days. Then, explants were transferred to shoot induction medium consisting of MS with 30 g/L sucrose, 2 mg/L *trans*-zeatin riboside, 0.1 mg/L indole-3-acetic acid (IAA), 20 mg/L hygromycin, 250 mg/L carbenicillin, and 8 g/L agar agar for 4–6 weeks. Explants with shoot buds were moved to shoot elongation medium consisting of MS with 30 g/L sucrose, 1 mg/L *trans*-zeatin riboside, 0.1 mg/L IAA, 20 mg/L hygromycin, 250 mg/L carbenicillin, and 8 g/L agar agar. Tomato shoots of about 2 cm height were cut and transferred to rooting medium consisting of MS with 30 g/L sucrose, 1 mg/L IAA, 10 mg/L hygromycin, 250 mg/L carbenicillin, and 8 g/L agar agar. Rooted plants were transferred into plastic pots containing potting mixture (Hanarum, Minong Fertilizer, Korea) and kept in a growth room maintained at 24°C and a 16 h light/8 h dark cycle.

### Nucleic Acid Extraction and Molecular Characterization

Genomic DNA (gDNA) was extracted from leaf samples of putative transgenic plants by the cetyltrimethylammonium bromide (CTAB) method (Porebski et al., 1997). gDNA was quantified by NanoDrop spectrophotometer (Nanodrop Technology, Inc., Wilmington, DE, USA) and diluted to 100 ng/μL. The status of putative transgenic plants was confirmed by PCR using *Hpt* (*Hygromycin phosphotransferase*) and *Cas9* gene-specific primers (**Table 1**). PCR conditions were as follows: 94°C for 5 min, followed by 34 cycles of denaturing at 94°C for 30 s, annealing from 55°C for 30 s, 72°C extension for 30 s, and then final extension at 72°C 5 min. Total RNA was extracted from young leaf tissues using an MG RNAzol kit according to the manufacturer’s instructions (MGmed, Seoul, Korea). The integrity and concentration of the total RNA were analyzed on a 1% agarose gel and a NanoDrop ND-1000 spectrophotometer (Thermo Scientific, Wilmington, USA), respectively. One microgram of total RNA was used to synthesize complementary DNA (cDNA) using an EasyScript Reverse Transcriptase kit (TransGen, Beijing, China) with oligo(dT) primers. The resulting cDNAs were used for further expression analysis. RT-PCR was performed as described for gene confirmation using 1 µL cDNA as a template.

**Table 1:** List of primers used in the present study.

Name	Primer sequence (5′ to 3′)	Amplicon size (bp)	Purpose
Sl4E_F_2	ACACTATGGTCCAAACAGTTCTTAT	330	Mutation detection
Sl4E_R_2	AACTGCTTGGGGAAGCTCAC	330	
SlPDS275_F	TGCTTCTCAACATAAATCTTGACAAAGAGAAGGA	275	
SlPDS275_R	CAAACCAAACCTTTAAAGGCCCCAAGT	275	
HygR_F	GCGAAGAATCTCGTGCTTTC	209	Transgene confirmation
HygR_R	CAACGTGACACCCTGTGAAC	209	
Cas9_pHSE_F	ATCCAATCTTCGGCAACAT	484	
Cas9_pHSE_R	TTATCCAGGTCATCGTCGTAT	484	
PDSgRNA_F	ATTGCTGTTAACTTGAGAGTCCA	–	sgRNA cloning
PDSgRNA_R	AAACTGGACTCTCAAGTTAACAG	–	
eIF4EgRNA_F	ATTGAGTTGAAGGCCGCCGATGG	–	
eIF4EgRNA_R	AAACCCATCGGCGGCCTTCAACT	–	

### Mutation Detection

The transgenic plants were genotyped for mutation detection using primers flanking sgRNA target regions. PCR products were purified using a LaboPass PCR clean-up kit (Cosmo Genetech, Seoul, Korea). The purified amplicons were also cloned into the TA cloning vector, pMD20-T (Mighty TA-cloning kit, TAKARA, Shiga, Japan), according to the manufacturer’s instructions, and positive colonies were selected by blue/white colony selection. Plasmids were extracted from least five positive clones and sequenced using M13F and M13R primers at Bionics (Seoul, Korea). To identify CRISPR/Cas9 induced mutations, DNA sequence alignments were performed using Lasergene’s SeqMan program (DNASTAR, Madison, WI, USA). Mutations in E_1_ progeny was detected by directly sequencing the PCR amplicons of the target region as well as by sequencing positive TA clones.

### Virus Inoculations

For virus inoculum preparation, frozen stocks of TEV-HAT and PepMoV-Vb1, which were stored at –80°C, were used to inoculate 3-week-old *Nicotiana benthamiana* plants. Frozen inocula were ground in 0.1 M potassium phosphate buffer (pH 7.0), mixed with 400-grit carborundum, and rubbed on the lower leaves of *N. benthamiana*. After 10–20 min of inoculation, leaves were washed with distilled water (Hull, 2009). To inoculate the tomato plants, infected *N. benthamiana* leaves were collected and inocula were prepared as mentioned above. Tomato plants with two fully expanded leaves were used for viral inoculation. Two pairs of cotyledons were inoculated; inoculated and non-inoculated control plants (mock) were grown in a growth chamber (16 h light and 8 h night under white fluorescent light). To ensure viral infection, plants were reinoculated 7 days after the first inoculation.

### Evaluation of Resistance to Potyviruses

After viral inoculation, plants were monitored regularly for the appearance of symptoms. Leaf tissue was tested for the presence of virus using double-antibody sandwich enzyme-linked immunosorbent assay (DAS-ELISA), according to the manufacturer’s instructions (Agdia, Inc. Elkhart, IN, USA). Virus accumulation was tested at 7/20 days post inoculation (DPI). DAS-ELISA was performed to detect the accumulation of the coat protein (CP) of TEV or PepMoV. Three replicates of inoculated and upper non-inoculated leaves of E_1_ lines were used for ELISA analysis. Absorbance of samples at 405 nm was measured using a microplate reader (Biotek, VT, USA). The statistical significance of the data was performed with Student’s t-test using Microsoft Excel 2016 (Microsoft, Redmond, WA, USA).

## Results

### Generation and Characterization of Genome-Edited Tomato Plants

**Figure 2:**
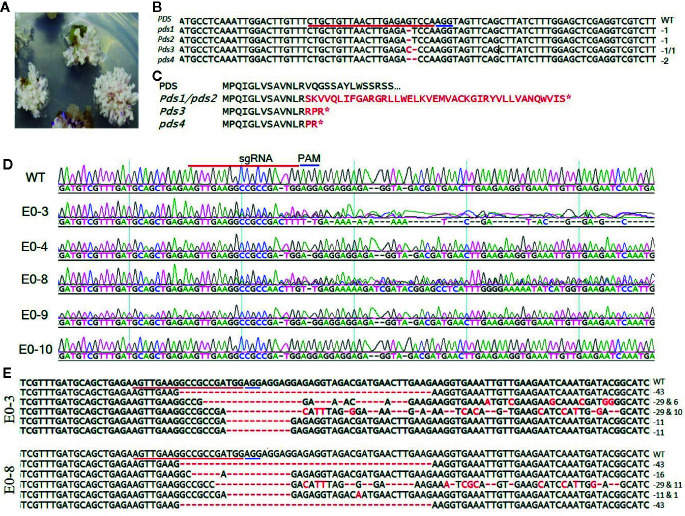
Sequence analysis of the *PDS*- and *eIF4E1*-targeted plants. **(A)** *PDS*-targeted explants showing the photobleached phenotype. **(B)** Mutation detection in *PDS*-targeted shoots by direct sequencing of PCR product in target region. **(C)** Predicted amino acid sequence alignment of *PDS* genes from genome-edited plants. Mutations causing early stop codons or changes in protein sequence are indicated with star symbol. **(D)** Representative chromatograms produced by direct sequencing of PCR products, displayed for sequence alignment of the mutated *eIF4E1* genes from the E0-3, E0-4, E0-8, E0-9, and E0-10 transgenic line. All transgenic lines with exception of the E0-10 showed mixed peaks in the target region due to their varied indels as compared with the wild-type sequence (WT) and are regarded as CRISPR/Cas9-induced mutant lines. **(E)** DNA sequences of mutated *eIF4E1* in the E0-3 and E0-8 line. The number of mutations of each type revealed by random sequencing of TA clones of PCR products. DNA deletions are denoted by red dashes, and deletion sizes (in nucleotides) are marked on the right side of the sequence. The sgRNA target sequence is underlined in red and the PAM motif in blue.

To develop tomato plants with edited *PDS* and *eIF4E1* genes, we delivered the pHSE401-PDS and pHSE401-eIF4E1 CRISPR/Cas9 constructs harboring the respective sgRNAs into *S. lycopersicum* cv. Micro-Tom *via Agrobacterium* transformation. We transferred putatively genome-edited shoots regenerated from the callus to shoot elongation medium and allowed them to elongate, and then cut elongated shoots and transferred them to rooting medium to encourage root formation. We observed the photobleached phenotype in four out of 113 *PDS*-edited explants transformed with the pHSE401-PDS construct (**Figure 2A**), demonstrating about 3.5% gene-editing efficiency of the *PDS* gene with both copies expected to be edited. However, the *PDS*-edited, photobleached shoots failed to develop into rooted shoots (**Figure 2A**). After sampling the photobleached shoots of the *PDS*-edited explants and putative *eIF4E1*-edited plants, we confirmed the integration of the transfer DNA (T-DNA) by genomic DNA PCR of the samples using *Cas9*- and *Hpt*-specific primers (**Supplementary Figure S1A**). We obtained PCR products with the expected amplicon sizes from the photobleached shoots, demonstrating the successful integration of *Cas9* and *Hpt* (**Supplementary Figure S1A**).

We also regenerated 22 putative *eIF4E1*-edited transgenic plants, which we tested for the presence of the transgenes, the *Cas9* and *Hpt* genes by PCR using transgene-specific primers (**Supplementary Figure S1B**). Sixteen of the 22 plants contained both *Cas9* and *Hpt*, with a transformation efficiency of 72%. Furthermore, RT-PCR results confirmed the expression of the *Cas9* gene in all the PCR positive plants (**Supplementary Figure S1C**). No morphological changes were observed in *eIF4E1*-edited plants.

### Confirmation of CRISPR/Cas9-Induced Mutations

To confirm that the CRISPR/Cas9 editing had introduced mutations in the *PDS* gene, we performed PCR amplification of the target region from the albino tomato mutants using primers (**Table 1**) that flanked the sgRNA target, and then sequenced the PCR products by Sanger sequencing. Sequence analysis showed two types of sequence variations: one- and two-nucleotide deletions with breakpoints 3 bp upstream of the protospacer-adjacent motif (PAM) sequence (**Figure 2B**). These deletions resulted in frameshift mutations causing early stop codons, preventing the expression of functional PDS protein (**Figure 2C**). Similarly, we identified CRISPR/Cas9-induced mutations in *eIF4E1* by sequencing the PCR amplicons of the sgRNA target-flanking region from the genomic DNA of 16 Cas9-positive tomato lines. Sequencing of the sgRNA target region revealed no mutant homozygous lines among the E_0_ plants; 15 of the 16 putative transgenic plants showed mixed sequence peaks at the sgRNA target site compared to the wild-type target sequence (**Figure 2D**), indicating that a mixture of mutant (reflective of CRISPR-Cas9-induced mutation) and wild-type alleles of *eIF4E1* were present. A single plant, E0-10 (**Figure 2D**) showed sequence peaks similar to that of wild-type target sequence, indicating no editing the in target region despite showing expression of the Cas9 gene. To further characterize the mutations in the E_0_ plants, we randomly selected the E0-3 and E0-8 transgenic lines for further investigation by TA cloning and sequencing of the target region. The sequencing results for the TA clones from E0-3 and E0-8 revealed four different alleles of *eIF4E1* (**Figure 2E**), with indels and/or substitutions at various positions both in proximity to the sgRNA target and outside the immediate sgRNA target region. The presence of a mixture of different mutant alleles in each E_0_ line suggested that active somatic mutation was occurring in the edited plants. Therefore, we advanced selected E_0_ plants to the E_1_ and E_2_ generations to create homozygous mutant lines.

### Inheritance of CRISPR/Cas9 Induced Mutations

**Figure 3:**
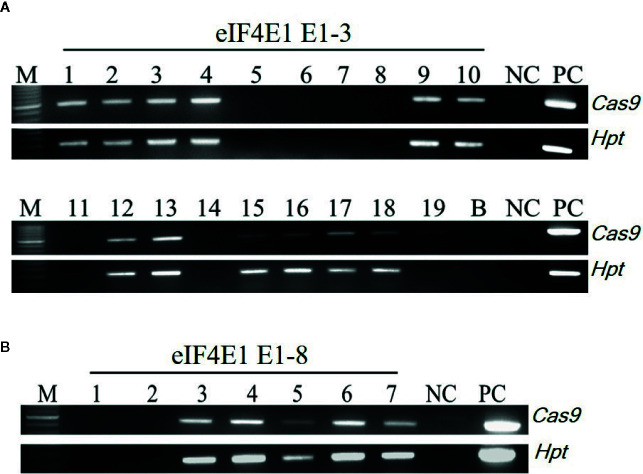
Transgene screening of *eIF4E1*-edited E_1_ progeny. **(A, B)** Transgene screening of *eIF4E1*-targeted E_1_ progeny of E0-3 (**A**; 19 progeny) and E0-8 (**B**; 7 progeny) by genomic DNA PCR. NC, wild type (negative control); PC, *Cas9* plasmid (positive control), *Hpt*, Hygromycin phosphotransferase marker gene.

Among the *eIF4E1* mutants, we selected the E0-3 and E0-8 plants from which to generate E_1_ lines. First, we allowed the E0-3 and E0-8 plants to self-pollinate to produce E_1_ lines. We then extracted gDNA from leaf samples of the E_1_ progeny and tested these for the presence of transgene by PCR with transgene-specific primers. Among 19 plants derived from E0-3, 13 carried transgenes (1, 2, 3, 4, 9, 10, 12, 13, 15, 16, 17, 18, and 19) and 6 did not (5, 6, 7, 8, 11, and 14), and among seven plants from the E0-8 plant, five carried transgenes (3, 4, 5, 6, and 7) and two did not (1 and 2) (**Figure 3**), indicative of the transgene-heterozygous status of the E_0_ plants.

**Figure 4:**
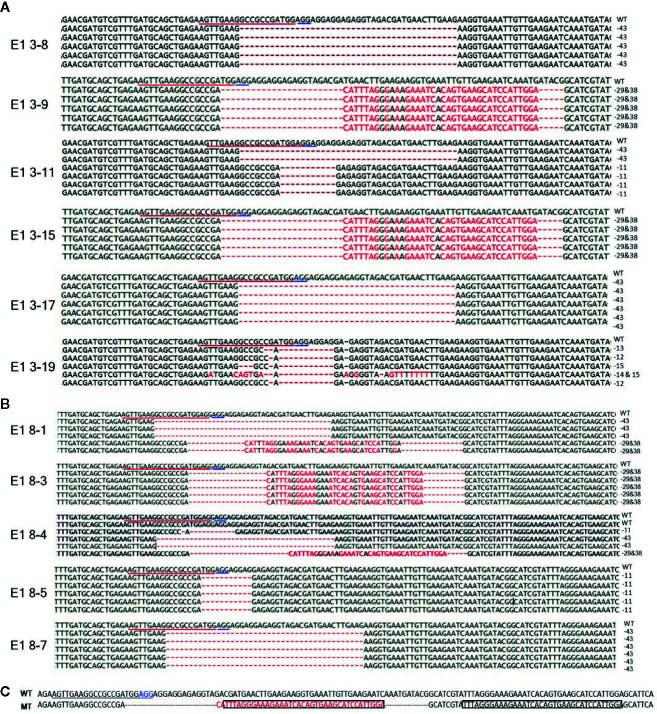
Genome-edited sequences of *eIF4E1* from E_1_ transgenic lines. **(A, B)** DNA sequences of the *eIF4E1* mutant lines derived from E0-3 **(A)** and E0-8 **(B)**. **(C)** Insertion of a 38-bp repeat sequence (boxed) from sgRNA adjacent region. The sgRNA target sequence is underlined in red and the PAM motif in blue. WT, wild type; MT, mutant lines (E1 3-9, E1 3-15, E1 8-1, and E1 8-3).

To reveal sequence variations of E_1_ plants, we performed TA cloning and sequencing of amplicons of the target region in E_1_ plants derived from lines E0-3 and E0-8. New mutation patterns were evident in the E_1_ lines as compared to the E_0_ plant, and two lines (E1 3-8 and E1 3-17) derived from E0-3 were homozygous for the expected 43-bp deletion (**Figure 4A**). The E1 3-11 line was biallelic, carrying two deletions of 43 and 11 bp; two other lines (E1 3-9 and 3-15) showed a 29-bp deletion and a tandem insertion of 38 bp of adjacent repeat sequence, respectively (**Figure 4C**); and the E1 3-19 line carried mixed mutations, with deletions of the unexpected sizes of 12, 13, and 15 bp (**Figure 4A**). The three lines (E1 8-3, 8-5, 8-7) derived from the E0-8 plant were homozygous for the expected 11-bp, 43-bp, and 29-bp deletions and 38-bp insertion of a repeat sequence (**Figures 4A, C** **)**. The E1 8-1 line was biallelic, with a 43-bp deletion and a 29-bp deletion and 38-bp repeat-sequence insertion, whereas the E1 8-4 line had mixed mutations along with a wild-type allele (**Figure 4B**). The presence of new mutation patterns in E_1_ lines that differed from those in the E_0_ plants suggested that active somatic mutation was occurring in the E_0_ plants (**Figures 2E** and **4**). Notably, one line, E1 3-8, had a homozygous edited *eIF4E1* gene but no T-DNA. Overall, several homozygous and biallelic lines were recovered in the E_1_ generation (**Table 2**), along with a smaller proportion of mosaic mutants carrying different allelic variants (**Table 2**).

**Table 2:** Summary of eIF4E1 CRISPR/Cas9‐induced mutations in E_1_ lines.

Line	Generation	Mutants analyzed	Mutant type	Number of plants	Percentage
E1 3	E_1_	19	Homozygous	7	36.8%
			Biallelic	8	42.1%
			Mosaic	4	21.0%
E1 8	E_1_	7	Homozygous	4	57.1%
			Biallelic	2	28.5%
			Mosaic	1	14.2%

### Potyvirus Resistance in *eIF4E1* Edited Plants

**Figure 5:**
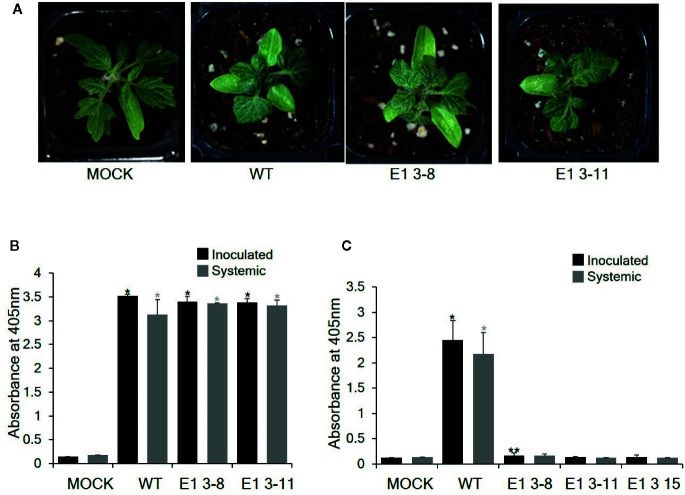
Resistance analysis of *eIF4E1* mutant lines challenged with potyviruses. **(A)** Phenotypes of *eIF4E1* mutant lines infected with TEV at 7 DPI. **(B, C)** Analysis of the accumulation of TEV-HAT **(B)** and PepMoV **(C)** in inoculated and systemic leaves by DAS-ELISA. Error bars indicate mean values of replicates ± SD. Asterisks indicate significant differences (*P < 0.01; **P < 0.05) between ‘mock′ and ‘treatments′ according to Student’s t‐test.

To investigate whether these CRISPR/Cas9-derived *eIF4E1* mutants conferred resistance to TEV, we inoculated E1 3-8 (homozygous line) and E1 3-11 (biallelic line) plants with TEV (**Figure 5**). TEV symptoms appeared as early as 7 DPI; the wild-type plant showed typical TEV symptoms, including vein clearing and several small chlorotic spots in the leaves. Both *eIF4E1* mutant lines showed similar symptoms (**Figure 5A**). To confirm virus infection, we performed DAS-ELISA analysis using inoculated lower and uninoculated upper leaves. We detected similar high amounts of virus coat protein accumulation in the E1 3-8 and E1 3-11 lines, indicating that the *eIF4E1*-edited lines were susceptible to TEV-HAT (**Figure 5B**). We next assessed whether CRISPR/Cas9 *eIF4E1* mutants could confer resistance to another potyvirus, PepMoV, by challenging three E_1_ mutant lines (E1 3-8, E1 3-11, and E1 3-15) with PepMoV. PepMoV coat protein accumulated to a high level in both the inoculated and uninoculated upper leaves of the susceptible wild-type control (**Figure 5**), whereas no coat protein accumulated in any of the three mutant lines in either the inoculated lower or uninoculated systemic leaves, confirming that the *eIF4E1*-edited lines were resistant to PepMoV (**Figure 5C**).

## Discussion

Here we subjected the *eIF4E1* gene to CRISPR/Cas9 gene editing with the aim of creating potyvirus resistance in *S. lycopersicum* Micro-Tom, as recessive resistance genes are considered to be confer more durable resistance than dominant resistance (*R*) genes (Kang et al., 2005a; Borrelli et al., 2018). In this study, we developed CRISPR/Cas9-edited tomato *PDS* and *eIF4E1* mutants through *Agrobacterium*-mediated delivery of CRISPR/Cas9 and sgRNA reagents. Sequencing of the *PDS* gene target region revealed that DNA double-strand edits occurred 3 bp upstream of the PAM site, resulting in indels of one or two nucleotides in *PDS*, as reported previously (Garneau et al., 2010; Jinek et al., 2012). All the indel mutations led to early stop codons, causing loss of *PDS* gene function. In the *eIF4E-*edited lines, indels occurred at various positions near the sgRNA target and in some cases extending beyond the sgRNA target and PAM region, creating premature stop codons and truncated eIF4E1 proteins (**Supplementary Data File 1**). Notably, we observed a 38-bp insertion of a repeat sequence as well as a 29-bp deletion in several of the gene-edited lines, E1 3-9, E1 3-15, E1 8-1, and E1 8-3; the mechanism leading to the insertion of this adjacent repeat sequence will require further investigation.

Unlike *PDS*-targeted photobleached plants, the *eIF4E1*-targeted mutant plants in the E_0_ generation were chimeric, with mixtures of different allelic variations, and the E_1_ generation comprised a mixture of homozygous, biallelic, and mosaic mutants. Increasing evidence suggests that transgenic plants can be chimeric in the E_0_ generation. For example, chimeric lines have also been reported in T_0_ (E_0_) genome-edited Arabidopsis, tomato, rice, and soybean (Brooks et al., 2014; Feng et al., 2014; Ma et al., 2015; Pan et al., 2016; Pyott et al., 2016; Liu et al., 2020). One study identified only three non-mosaic mutants among nearly 300 lines of Arabidopsis created by editing of the gene *RRP42* (Yan et al., 2017). The mosaicism of the E_1_ mutants could then be due to the fact that in some edited lines, one or more wild-type alleles might escape detection in the E_0_ generation but then be detected in later generations (Brooks et al., 2014; Zhang et al., 2014; Zhang et al., 2019; Liu et al., 2020). Furthermore, other new alleles created from late-arising chimeric tissues might also be overlooked in E_0_ plants, resulting in different flowers carrying different alleles (Brooks et al., 2014). In agreement with this contention, in the present study, some of the E_1_ plants produced by gene editing of *eIF4E1* were chimeric, and furthermore mutations different from those identified in E_0_ plants were observed in E_1_ plants.

Notwithstanding these complications, we advanced the E_0_ plants carrying diverse alleles of the target gene to the next generation, and selected E_1_ lines carrying homozygous alleles from segregating populations, as reported in previous studies (Xu et al., 2015; Chandrasekaran et al., 2016; Pyott et al., 2016). Accordingly, sequence analysis of the E_1_ lines identified both homozygous and biallelic *eIF4E1* mutants, both of which would be expected to stably pass on their mutant status to their descendants (Chandrasekaran et al., 2016), consistent with this, homozygous mutant alleles were stably inherited in the E_2_ progeny.

Previous studies in pepper suggest that eIF4E is a key factor in the interaction between viruses and their hosts. Mutations in *eIF4E* cause conformational shifts in the encoded proteins, interrupting the interaction between VPg and eIF4E and conferring plant resistance to the virus at the cellular level (Kang et al., 2005a). This implied that site-directed mutagenesis could be used to create dominant negative mutations or targeted silencing of host genes (Azevedo et al., 2002; Kang et al., 2005b; Kang et al., 2007). We therefore assessed our eIF4E-edited E_1_ lines, with or without the *Cas9* transgene, for resistance to potyviruses. Edited plants carrying mutations in *eIF4E1* showed resistant to PepMoV. Consistent with this, we did not detect accumulation of coat protein in either inoculated or uninoculated leaves. However, E_1_ plants carrying mutations in *eIF4E1* showed typical TEV symptoms, and ELISA analysis confirmed the presence of systemic infection. Viral coat protein accumulated to high levels in both inoculated and uninoculated upper leaves of the homozygous E1 3-8 and biallelic E1 3-11 lines. These results contrast with the findings of an earlier study indicating that transgenic tomato plants overexpressing a recessive resistance allele of *eIF4E (pvr1*) from *Capsicum chinense* showed dominant resistance to TEV (Kang et al., 2007). The transgenic expression of the *Capsicum* *eIF4E* mutant allele was suggested to perturb the interactions required for viral susceptibility in a heterologous host system (Kang et al., 2007). However, the susceptibility of the edited plants to TEV in tomato that we observed here could be due to the redundant activities of *eIF4E* homologs (*eIF4E1* and *eIF4E2*) coupled with the absence of a dominant negative allele whose protein product could interfere with the endogenous wild‐type proteins and inhibit viral infection (Kang et al., 2007). Furthermore, an *eIF4E1*-knockout tomato plant selected from a TILLING population was reported to be susceptible to potyviruses because TEV could utilize either *eIF4E1* or *eIF4E2* for its replication in tomato (Gauffier et al., 2016). Thus, development of a TEV virus-resistant tomato line is hindered by the gene redundancy of *eIF4E* homologs. *eIF4E1* and *eIF4E2* double-knockout plants would be expected to show a broad-spectrum resistance to a wide range of potyviruses (Gauffier et al., 2016); however, care should be taken to ensure that mutations in both candidate genes do not impair the plant growth and development, as often occurs with double mutants (Mazier et al., 2011; Gauffier et al., 2016).

## Conclusion

We used CRISPR/Cas9 gene editing to induce mutations in the tomato *eIF4E1* gene by *A. tumefaciens*-mediated transformation. Evaluation of the mutation and inheritance patterns of this gene in the E_0_ and later generations by sequencing revealed high proportions of chimeric mutant lines in the E_0_ generation and of homozygous and biallelic mutants of *eIF4E1* in the E_1_ generation. The CRISPR/Cas9-mediated gene mutations were stably transmitted to the E_1_ and E_2_ descendants irrespective of the presence or absence of T-DNA. Although the presence of redundant *eIF4E* homologs apparently precluded TEV resistance in the *eIF4E1* genome-edited Micro-Tom plants, an edited *eIF4E1* with an early stop codon conferred resistance to PepMoV. Thus, our system enabled successful CRISPR/Cas9 editing of tomato *eIF4E1*, resulting in resistance to a common viral pathogen of Solanaceae; further research is planned to obtain homozygous E_0_ plants and to extend this work to pepper (*Capsicum* species) to produce strains with broad-spectrum virus resistance.

## Data Availability Statement

The original contributions presented in the study are included in the article/**Supplementary Material**. Further inquiries can be directed to the corresponding author.

## Author Contributions

Y-JY and JV designed the constructs and performed the transformation. Y-JY, D-SK, JK, and H-EL characterized the transgenics and identified the mutants. Y-JY, J-HL and JV performed viral inoculations and ELISA. JV wrote the manuscript. B-CK, D-SK, JK, H-EL, and JV revised the manuscript. B-CK conceived and designed the research. All authors contributed to the article and approved the submitted version.

## Funding

This work was carried out with the support of the Cooperative Research Program for Agriculture Science & Technology Development (Project No. PJ01327801, PJ01327803), Rural Development Administration, Republic of Korea.

## Conflict of Interest

The authors declare that the research was conducted in the absence of any commercial or financial relationships that could be construed as a potential conflict of interest.
