# Supplementary material for: Genome Editing of eIF4E1 in Tomato Confers Resistance to Pepper Mottle Virus
Source: Front Plant Sci. 2020 Jul 24;11:1098. doi: 10.3389/fpls.2020.01098 (PMC7396686; doi:10.3389/fpls.2020.01098)
Supplement: Supplementary file 1 [file DataSheet_1.docx]

**Supplementary data file1. CRISPR/Cas9 induced mutations in *eIF4E* genome edited lines.** Truncation of proteins due to early stop codons are indicated with star symbol. Changes in protein sequences due to frameshift mutation are indicated with red font.

>WT

maaaemertmsfdaaeklkaadggggevddeleegeiveesndtasylgkeitvkhplehswtfwfdnpttksrqtawgsslrnvytfstvedfwgaynnihhpsklimgadfhcfkhkiepkwedpvcanggtwkmsfskgksdtswlytllamighqfdhgdeicgavvsvrakgekialwtknaanetaqvsigkqwkqfldysdsvgfifhddakrldrnaknrytv*

>E1 3-11(-11)

maaaemertmsfdaaeklkaaergrr*t*rr*nc*rik*ygivfrernhseasigafmdflv**pyh*istnclgkltskclhflhc*rflgclq*ypspkqvnygsrlslf*aqn*akvgrscmcqwrdveneffeg*i*yqlavyaagndwtsirswr*nlwssc*cpg*grknsfvdqecck*nssg*hw*ameavsrlq*fgwlhisrrckearqkcqeslhrix

>E1 3-11(-43)

maaaemertmsfdaaeklkkvkllknqmirhri*gkksq*sihwsihglfglitlplnldkllgeahfemstlspllkifgvltiisitqas*lweqtfivlstklsqsgkilyvpmegrgk*vfrrvnlipagcircwq*ldinsimemkfveqllvsglrekk*lcgprmlqmkqlrlalvsngssf*itvirlasyfttmqrgstemprivtpyx

>E1 3-9 & E1 3-15 (-29 & 38)

maaaemertmsfdaaeklkaadi*gkksq*sihwsivfrernhseasigafmdflv**pyh*istnclgkltskclhflhc*rflgclq*ypspkqvnygsrlslf*aqn*akvgrscmcqwrdveneffeg*i*yqlavyaagndwtsirswr*nlwssc*cpg*grknsfvdqecck*nssg*hw*ameavsrlq*fgwlhisrrckearqkcqeslhrix

>E1 3-19 (-12)

maaaemertmsfdaaeklkaarevddeleegeiveesndtasylgkeitvkhplehswtfwfdnpttksrqtawgsslrnvytfstvedfwgaynnihhpsklimgadfhcfkhkiepkwedpvcanggtwkmsfskgksdtswlytllamighqfdhgdeicgavvsvrakgekialwtknaanetaqvsigkqwkqfldysdsvgfifhddakrldrnaknrytv*

>E1 3-19 (-15)

maaaemertmsfdaaeklkarevddeleegeiveesndtasylgkeitvkhplehswtfwfdnpttksrqtawgsslrnvytfstvedfwgaynnihhpsklimgadfhcfkhkiepkwedpvcanggtwkmsfskgksdtswlytllamighqfdhgdeicgavvsvrakgekialwtknaanetaqvsigkqwkqfldysdsvgfifhddakrldrnaknrytv*

>E1 3-19 (-13 & 15)

maaaemertmsfdaaekmnseegvsfflkkvkllknqmirhri*gkksq*sihwsihglfglitlplnldkllgeahfemstlspllkifgvltiisitqas*lweqtfivlstklsqsgkilyvpmegrgk*vfrrvnlipagcircwq*ldinsimemkfveqllvsglrekk*lcgprmlqmkqlrlalvsngssf*itvirlasyfttmqrgstemprivtpyx

>E1 E1 3-8 & 3-17 (-43)

maaaemertmsfdaaeklkkvkllknqmirhri*gkksq*sihwsihglfglitlplnldkllgeahfemstlspllkifgvltiisitqas*lweqtfivlstklsqsgkilyvpmegrgk*vfrrvnlipagcircwq*ldinsimemkfveqllvsglrekk*lcgprmlqmkqlrlalvsngssf*itvirlasyfttmqrgstemprivtpyx

>E1 8-1 (-43)

maaaemertmsfdaaeklkkvkllknqmirhri*gkksq*sihwsihglfglitlplnldkllgeahfemstlspllkifgvltiisitqas*lweqtfivlstklsqsgkilyvpmegrgk*vfrrvnlipagcircwq*ldinsimemkfveqllvsglrekk*lcgprmlqmkqlrlalvsngssf*itvirlasyfttmqrgstemprivtpyx

>E1 8-1 (-29 & 38)

maaaemertmsfdaaeklkaadi*gkksq*sihwsivfrernhseasigafmdflv**pyh*istnclgkltskclhflhc*rflgclq*ypspkqvnygsrlslf*aqn*akvgrscmcqwrdveneffeg*i*yqlavyaagndwtsirswr*nlwssc*cpg*grknsfvdqecck*nssg*hw*ameavsrlq*fgwlhisrrckearqkcqeslhrix

>E1 8-3 (-29 & 38)

maaaemertmsfdaaeklkaadi*gkksq*sihwsivfrernhseasigafmdflv**pyh*istnclgkltskclhflhc*rflgclq*ypspkqvnygsrlslf*aqn*akvgrscmcqwrdveneffeg*i*yqlavyaagndwtsirswr*nlwssc*cpg*grknsfvdqecck*nssg*hw*ameavsrlq*fgwlhisrrckearqkcqeslhrix

>E1 8-4 (-12)

maaaemertmsfdaaeklkaarevddeleegeiveesndtasylgkeitvkhplehswtfwfdnpttksrqtawgsslrnvytfstvedfwgaynnihhpsklimgadfhcfkhkiepkwedpvcanggtwkmsfskgksdtswlytllamighqfdhgdeicgavvsvrakgekialwtknaanetaqvsigkqwkqfldysdsvgfifhddakrldrnaknrytv*

>E1 8-4 (-43)

maaaemertmsfdaaeklkkvkllknqmirhri*gkksq*sihwsihglfglitlplnldkllgeahfemstlspllkifgvltiisitqas*lweqtfivlstklsqsgkilyvpmegrgk*vfrrvnlipagcircwq*ldinsimemkfveqllvsglrekk*lcgprmlqmkqlrlalvsngssf*itvirlasyfttmqrgstemprivtpyx

>E1 8-4 (-29 & 38)

maaaemertmsfdaaeklkaadi*gkksq*sihwsivfrernhseasigafmdflv**pyh*istnclgkltskclhflhc*rflgclq*ypspkqvnygsrlslf*aqn*akvgrscmcqwrdveneffeg*i*yqlavyaagndwtsirswr*nlwssc*cpg*grknsfvdqecck*nssg*hw*ameavsrlq*fgwlhisrrckearqkcqeslhrix

>E1 8-5 (-11)

maaaemertmsfdaaeklkaaergrr*t*rr*nc*rik*ygivfrernhseasigafmdflv**pyh*istnclgkltskclhflhc*rflgclq*ypspkqvnygsrlslf*aqn*akvgrscmcqwrdveneffeg*i*yqlavyaagndwtsirswr*nlwssc*cpg*grknsfvdqecck*nssg*hw*ameavsrlq*fgwlhisrrckearqkcqeslhrix

>E1 8-7(-43)

maaaemertmsfdaaeklkkvkllknqmirhri*gkksq*sihwsihglfglitlplnldkllgeahfemstlspllkifgvltiisitqas*lweqtfivlstklsqsgkilyvpmegrgk*vfrrvnlipagcircwq*ldinsimemkfveqllvsglrekk*lcgprmlqmkqlrlalvsngssf*itvirlasyfttmqrgstemprivtpyx
