# Supplementary figures and images for: Genome Editing of eIF4E1 in Tomato Confers Resistance to Pepper Mottle Virus
Source: Front Plant Sci. 2020 Jul 24;11:1098. doi: 10.3389/fpls.2020.01098 (PMC7396686; doi:10.3389/fpls.2020.01098)

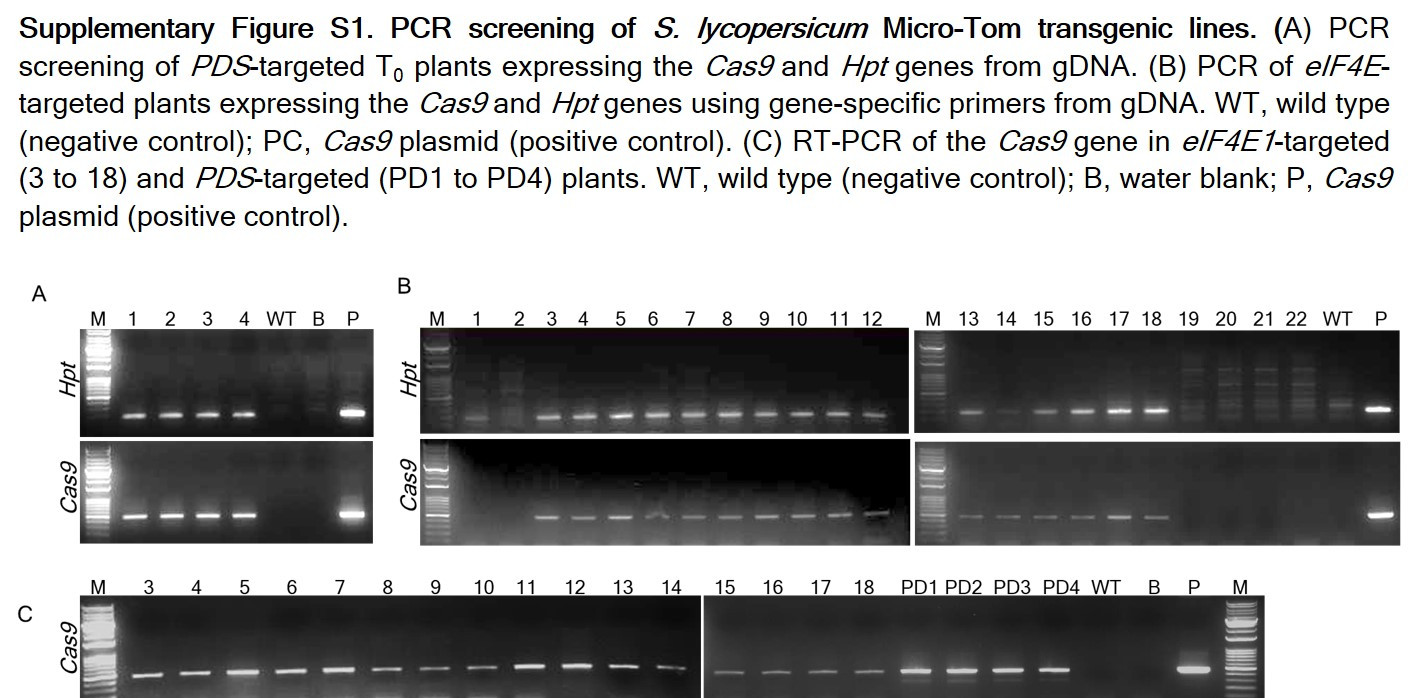

Supplement: Supplementary file 2 [file Image_1.jpg]
